# Current status of molecular diagnostics for lung cancer

**DOI:** 10.37349/etat.2024.00244

**Published:** 2024-06-27

**Authors:** Evgeny N. Imyanitov, Elena V. Preobrazhenskaya, Sergey V. Orlov

**Affiliations:** IRCCS Istituto Romagnolo per lo Studio dei Tumori (IRST) "Dino Amadori", Italy; ^1^Department of Tumor Growth Biology, N.N. Petrov Institute of Oncology, 197758 St.-Petersburg, Russia; ^2^Department of Clinical Genetics, St.-Petersburg State Pediatric Medical University, 194100 St.-Petersburg, Russia; ^3^I.V. Kurchatov Complex for Medical Primatology, National Research Centre “Kurchatov Institute”, 354376 Sochi, Russia; ^4^Department of Oncology, I.P. Pavlov St.-Petersburg State Medical University, 197022 St.-Petersburg, Russia

**Keywords:** Lung cancer, mutations, fusions, predictive markers, therapy

## Abstract

The management of lung cancer (LC) requires the analysis of a diverse spectrum of molecular targets, including kinase activating mutations in *EGFR*, *ERBB2* (*HER2*), *BRAF* and *MET* oncogenes, *KRAS* G12C substitutions, and *ALK*, *ROS1*, *RET* and *NTRK1-3* gene fusions. Administration of immune checkpoint inhibitors (ICIs) is based on the immunohistochemical (IHC) analysis of PD-L1 expression and determination of tumor mutation burden (TMB). Clinical characteristics of the patients, particularly age, gender and smoking history, significantly influence the probability of finding the above targets: for example, LC in young patients is characterized by high frequency of kinase gene rearrangements, while heavy smokers often have *KRAS* G12C mutations and/or high TMB. Proper selection of first-line therapy influences overall treatment outcomes, therefore, the majority of these tests need to be completed within no more than 10 working days. Activating events in MAPK signaling pathway are mutually exclusive, hence, fast single-gene testing remains an option for some laboratories. RNA next-generation sequencing (NGS) is capable of detecting the entire repertoire of druggable gene alterations, therefore it is gradually becoming a dominating technology in LC molecular diagnosis.

## Introduction

Lung cancer (LC) often serves as the best example of opportunities provided by precision medicine [1–6]. More than half of lung adenocarcinomas have a druggable oncogenic dependency, which can be detected by conventional genetic analysis and efficiently targeted by a spectrum of relevant drugs. The progress in the identification of mutated kinases and the development of associated therapies resulted in a manifold improvement of patient outcomes. While the median life expectancy of patients with metastatic LC was below one year just two decades ago, this estimate currently exceeds 3 years for subjects with *EGFR*-mutated disease and approaches more than 7 years for *ALK*-driven carcinomas [7–13]. There are about a dozen different targets that require molecular testing, with each of them usually linked to several potentially efficient therapeutic compounds (Table 1). This review provides an update on the increasing complexity of the genetic analysis of LC and associated treatment decisions.

**Table 1:** Major achievements in clinical outcomes for patients with druggable genetic alterations

**Gene/Mutation**	**Drug**	**Study**	**Number of patients**	**Response rate**	**PFS**	**OS**	**Reference**	**Brief description of the study**
*EGFR* ex19del and L858R	Osimertinib	NCT02296125 (FLAURA)	279, treatment-naive	80%	18.9 months	38.6 months	[11]	Phase III, osimertinib vs. 1st-generation TKI
	Osimertinib + chemotherapy	NCT04035486 (FLAURA2)	279, treatment-naive	83%	29.4 months	Not reached	[14]	Phase III, osimertinib plus chemotherapy vs. osimertinib alone
	Erlotinib + ramucirumab	NCT02411448 (RELAY)	224, treatment-naive	76%	19.4 months	Not reached	[15]	Phase III, erlotinib plus ramucirumab vs. erlotinib alone
*EGFR* exon 20 insertions	Amivantamab	NCT02609776 (CHRYSALIS)	81, previously treated	40%	8.3 months	22.8 months	[16]	Phase I
*ERBB2* (*HER2*) mutations	Trastuzumab deruxtecan (T-DXd)	NCT04644237 (DESTINY-Lung02)	152, previously treated	49% (5.4 mg/kg); 56% (6.4 mg/kg)*	9.9 months (5.4 mg/kg); 15.4 months (6.4 mg/kg)*	19.5 months (5.4 mg/kg); not reached (6.4 mg/kg)*	[17]*	Phase II, comparison of two drug doses
*BRAF* V600 mutations	Dabrafenib + trametinib	NCT01336634	36, treatment-naive	64%	10.9 months	17.3 months	[18]	Phase II
	Encorafenib + binimetinib	NCT03915951	59, treatment-naive	75%	Not reached	Not reached	[19]	Phase II
*KRAS* G12C	Sotorasib	NCT04303780 (CodeBreak 200)	141, previously treated by chemotherapy and ICI	28%	5.6 months	10.6 months	[20]	Phase III, sotorasib vs. docetaxel
	Adagrasib	NCT03785249 (KRYSTAL-1)	112, previously treated by chemotherapy and ICI	43%	6.5 months	12.6 months	[21]	Phase I−II
*MET* exon 14 skipping mutations	Capmatinib	NCT02414139 (GEOMETRY)	28, treatment-naive	68%	12.4 months	20.8 months	[22]	Phase II
	Tepotinib	NCT02864992 (VISION)	164, treatment-naive	57%	12.6 months	21.3 months	[23]	Phase II
*ALK* fusions	Alectinib	NCT02075840 (ALEX)	152, treatment-naive	83%	34.8 months	Not reached	[24]	Phase III, alectinib vs. crizotinib
	Lorlatinib	NCT03052608 (CROWN)	149, treatment-naive	76%	Not reached (above 3 years)	Not reached	[25]	Phase III, lorlatinib vs. crizotinib
*ROS1* fusions	Crizotinib	NCT00585195 (PROFILE 1001)	53, previously treated and treatment-naive	72%	19.3 months	51.4 months	[26]	Phase I
	Entrectinib	NCT02097810 (STARTRK-1), NCT02568267 (STARTRK-2), EudraCT, 2012-000148-88 (ALKA-372-001)	168, previously treated (without TKI) and treatment-naive	68%	15.7 months	47.8 months	[27]	Phase I−II
	Repotrectinib	NCT03093116 (TRIDENT-1)	71, treatment-naive	79%	35.7 months	Not reached	[28]	Phase I−II
*RET* fusions	Pralsetinib	NCT04222972 (ARROW)	75, treatment-naive	72%	13.0 months	Not reached	[29]	Phase I−II
	Selpercatinib	NCT04194944 (LIBRETTO-431)	129, treatment-naive	84%	24.8 months	Not reached	[30]	Phase III, selpercatinib vs. pembrolizumab plus chemotherapy
*NTRK* fusions	Entrectinib	NCT02097810 (STARTRK-1), NCT02568267 (STARTRK-2), EudraCT, 2012-000148-88 (ALKA-372-001)	51, previously treated and treatment-naive	63%	28.0 months	41.5 months	[31]	Phase I−II
	Larotrectinib	NCT02576431 and NCT02122913	20, previously treated	73%	35.4 months	40.7 months	[32]	Phase I−II

* Significantly higher incidence of severe adverse event at higher dose of the drug. PFS: progression-free survival; OS: overall survival; ICI: immune checkpoint inhibitor; TKI: tyrosine kinase inhibitor

## The diversity of *EGFR* mutations

Tyrosine kinase inhibitor (TKI) sensitizing *EGFR* mutations were discovered via DNA analysis of tumor tissues obtained from responders to gefitinib or erlotinib [33]. It was immediately recognized that majority of these mutations are located within hot-spots. Approximately two thirds of these predictive mutations are in-frame deletions affecting exon 19 (ex19del), and about a third of *EGFR*-mutated LCs carry L858R substitution in exon 21. Ex19del mutations are all located within a narrow region of the gene and represented by several distinct variants [34]. A recent study revealed the most common deletion, E746_A750del, which accounts for more than a quarter of ex19del alterations, may render higher sensitivity to TKIs than some other mutations affecting *EGFR* exon 19 [35]. No other clinical studies have yet considered the diversity of these in-frame deletions, assuming that they have only minor differences with regard to their size and exact location.

L858R-mutated LCs consistently demonstrate less pronounced response to TKIs as compared to ex19del-driven tumors [36]. Importantly, L858R substitutions are particularly characteristic for elderly patients, therefore, age-related factors may compromise treatment trajectories [37]. Afatinib registration trials demonstrated that patients with *EGFR* ex19del mutations receiving targeted therapy in the first line have an overall survival (OS) advantage as compared to patients treated with TKI after chemotherapy. In fact, the opposite trend, i.e., the numerical OS advantage of the first-line chemotherapy, was observed in patients with *EGFR* L858R substitutions, although this difference was statistically non-significant [38]. A lower frequency of L858R versus ex19del could have contributed to the statistical conclusions of this and some other studies. The FLAURA trial has changed the standards of upfront treatment of *EGFR*-mutated LC by demonstrating that patients treated with osimertinib had longer OS as compared to subjects receiving gefitinib or erlotinib. It is rarely acknowledged that this advantage was seen only for ex19del-mutated LCs, while both study arms produced exactly the same outcomes for patients with L858R substitutions [11]. Furthermore, afatinib even outperformed osimertinib for L858R-mutated patients without brain metastases in a Japanese multicenter study [13]. It may be reasonable to consider ex19del- and L858R-driven LCs as distinct tumor entities. There are some treatment regimens demonstrating a promising outcome for patients with L858R mutations. For example, unlike single-agent gefitinib, erlotinib, afatinib or osimertinib, a second-generation EGFR TKI dacomitinib showed similar responses in patients with ex19del and L858R [39]. In contrast to the FLAURA trial [11], it revealed statistically significant OS advantage against gefitinib in patients with L858R mutation, while this superiority was less pronounced for ex19del-mutated LCs [40]. A combination of erlotinib plus ramucirumab has also produced similar response rates (RRs) and progression-free survival (PFS) in both ex19del and L858R patient subgroups [41]. The ongoing clinical study is intended to directly compare the efficacy of this combination versus osimertinib in previously untreated LC patients with *EGFR* L858R mutations [42]. The FLAURA2 investigation has demonstrated that the upfront administration of osimertinib plus chemotherapy is associated with significantly longer median PFS than osimertinib alone; the relative gain of PFS appeared to be numerically more recognizable for patients with L858R substitutions than for subjects with exon 19 deletions [14].

Exon 19 deletions and codon 858 substitutions account for approximately 80−90% of EGFR kinase-activating mutations located within exons 18−21. The remaining mutations are classified in literature as “rare”, “uncommon”, or “atypical”. The genetic lesions affecting exons 18, 19 and 21 compose a diverse spectrum of events, with most frequent substitutions affecting codons 709, 719, 768 and 861. Preclinical data suggest that second-generation EGFR TKIs have generally greater activity towards uncommon mutations [43, 44]. In line with this, afatinib is the only TKI which received FDA approval for the treatment of LC with atypical mutations [45]. Nevertheless, several clinical trials demonstrated significant activity of osimertinib towards rare *EGFR* mutations, therefore, this option is also included in the treatment guidelines [45–47]. Exon 19 in-frame insertions constitute approximately 1% of *EGFR* mutations and render tumor sensitivity to conventional EGFR inhibitors [48]. Other categories of *EGFR* exon 18, 19 and 21 mutations are exceptionally rare. For the time being, all above alterations are treated as a single entity. However, both laboratory data and clinical observations suggest that there are significant variations in drug sensitivity across this spectrum, with some of these genetic events apparently associated with an exceptional sensitivity to the old-generation drugs [43, 49–51].

Exon 20 insertions constitute an especial and highly diverse category of *EGFR* mutations [5, 45, 52]. They are observed in approximately 2% of lung carcinomas. *EGFR* A763_Y764insFQEA and D761_E762insEAFQ mutations are associated with tumor sensitivity to conventional EGFR inhibitors [45, 53]. The treatment of tumors harboring the remaining categories of mutations is highly complicated. Low-weight EGFR TKI poziotinib demonstrated 31% RR and 5.5 months median PFS in this subset of LC patients [54]. Another EGFR TKI, mobocertinib, also has shown moderate activity (RR: 28%; median PFS: 7.3 months) in a clinical setting, however, its initial approval was subsequently withdrawn due to lack of efficacy in a phase III study [55]. Although earlier studies on *EGFR*-mutated LC were focused mainly on small-molecule drugs, the clinical activity of amivantamab, a dual EGFR and MET inhibitor, calls for reconsideration of this concept. Amivantamab caused tumor responses in 40% of the analyzed patients, with median PFS approaching 8.3 months [16]. For the time being, amivantamab is the only drug approved for LC patients with *EGFR* exon 20 insertions. The diversity of exon 20 insertions remains underappreciated; it is likely that different categories of these mutations require somewhat different treatment [53, 54].

*EGFR* mutations were among the first genetic predictive markers included in the treatment guidelines. The knowledge of *EGFR* mutations emerged in the middle of the 1st decade of this century, well before the incorporation of next-generation sequencing (NGS) technologies into clinical routine. The discovery and clinical adoption of *EGFR* mutations led to the development of a number of diagnostic kits, which rely on allele-specific PCR and are capable of detecting the most common somatic variants. The utilization of these kits has become widespread, as they are compatible with the capacities of local hospital laboratories. It is self-explanatory that these kits cannot detect the entire spectrum of *EGFR* genetic lesions. First of all, presence of rare mutations rendering tumor responsiveness to conventional EGFR TKIs (gefitinib, erlotinib, afatinib, osimertinib, etc.) cannot be reliably excluded using mutation-specific PCR. Secondly, none of PCR kits properly account for exon 20 insertions, given that the corresponding laboratory protocols had been developed at the time when the frequency, repertoire and predictive significance of *EGFR* exon 20 alterations remained unclear. Therefore, the use of PCR kits for *EGFR* testing may need to be discouraged in forthcoming years [52, 53, 56, 57].

## Other hot-spot mutations accessible to conventional DNA-based testing

*BRAF* codon 600 substitutions were discovered via preplanned search for kinase-activating mutations [58]. These alterations are characteristic for several tumor types, including melanomas, colorectal cancers, thyroid malignancies, lung carcinomas, etc. *BRAF* V600 mutations occur in less than 2% of lung carcinomas, being more frequent in non-smokers and females (Table 2). Initial attempts to treat *BRAF*-mutated cancers by single-agent BRAF inhibitors revealed that tumors rapidly adapt to this therapy by activation of the MEK kinase. Current treatment guidelines suggest using doublet BRAF and MEK inhibition for the treatment of *BRAF*-driven malignancies. Combined utilization of BRAF and MEK antagonists produced high response rates in patients with advanced LC [19]. Data on overall survival for patients with *BRAF* codon 600 mutations suggest consistent but moderate gain in life expectancy [63, 64].

**Table 2:** Distribution of druggable genetic alterations in patients of different race, age, gender and smoking history

**Type of genetic alteration**	**Gene**	**Frequency**	**Race**	**Age**	**Gender**	**Smoking history**	**References**
Mutations	*EGFR*	10−20% in non-Asians, up to 60−70% in Asians	More common in Asians	L858R substitution is particularly common in elderly patients	More common in females	More common in non-smokers	[33, 37, 59]
	*ERBB2* (*HER2*)	2−3%		More common in young patients	More common in females	More common in non-smokers	[60–62]
	*BRAF* V600	1−2%		More common in elderly patients (?)	More common in females	More common in non-smokers	[62–65]
	*KRAS* G12C	10−15%				More common in smokers	[5, 66]
	*MET*	2−3%		More common in elderly patients	More common in females	More common in non-smokers	[67, 68]
Rearrangements	*ALK*	4−5%		More common in young patients	More common in females	More common in non-smokers	[69]
	*ROS1*	1−2%		More common in young patients	More common in females	More common in non-smokers	[69, 70]
	*RET*	2−4%		More common in young patients (?)	More common in females	More common in non-smokers	[71]
	*NTRK1*−*3*	0.2%		More common in young patients	More common in females	More common in non-smokers	[72, 73]

(?): not enough evidence. The blank cells indicate lack of association or absence of relevant information

*BRAF* V600 substitutions are a predominant type of somatic *BRAF* alterations in the majority of tumor types (melanoma, colorectal cancer, etc.), however, they account for less than a half of *BRAF* lesions in lung malignancies. There are several other *BRAF* hot-spot codons located in exon 11 (G464, G466, G469) or exon 15 (D594, G596, L597, K601), which are relevant to LC pathogenesis, however, they cannot be efficiently targeted by currently available therapies, and, therefore, are not mandatory for LC molecular testing [63, 64].

*KRAS*, *NRAS* and *HRAS* nucleotide substitutions involving codons 12, 13, 59, 61, 146, etc. were among the first oncogene-activating events discovered in the early years of emergence of molecular oncology. *RAS* alterations are very frequent across different tumor types and, therefore, appear to be a promising molecular target. However, the development of *RAS* mutation-specific drugs is compromised by the diversity of *RAS* mutations, the small size of RAS proteins and their high affinity to a substrate molecule, GTP [74]. For the time being, only KRAS G12C inhibitors have been adopted into clinical practice. *KRAS* G12C substitution occurs at significant frequency in lung and colorectal carcinomas [74, 75]. In LC, *KRAS* G12C mutations are particularly characteristic for tumors induced by tobacco carcinogens, being detected in approximately 1 out of 6−7 smokers [65, 66]. Smoking-associated LCs usually contain an increased number of carcinogen-induced mutations, and, therefore, are highly antigenic and responsive to immune therapy. The efficacy of KRAS G12C targeted drugs was evaluated in LC patients, who had already received cytotoxic drugs and immune checkpoint inhibitors (ICIs). Sotorasib and adagrasib demonstrated moderate efficacy with regard to RRs (28% and 43%, respectively) and median PFS (5.6 months and 6.5 months, respectively) [20, 21]. A comparison of sotorasib with docetaxel revealed only small advantage for PFS and no statistically significant difference for OS [20]. Another KRAS inhibitor, MRTX1133, has been recently developed for targeting KRAS G12D mutations [76].

In addition to the direct targeting of RAS-mutated proteins, there are attempts to interfere with the consequences of KRAS activation. MEK inhibitors have a potential to inhibit all tumors with upstream activating events, be it receptor tyrosine kinase (RTK), *BRAF* or *RAS* mutation. Cancer cells respond to MEK down-regulation by autophagy, which serves as a therapy escape mechanism. Hydroxychloroquine, commonly known as Plaquenil, is a well-known inhibitor of autophagy. Several case reports have described the shrinkage of *RAS*-mutated tumors in pancreatic and colorectal cancer patients in response to a combined administration of hydroxychloroquine and MEK inhibitors [77, 78]. Despite these promising data, a small clinical trial failed to demonstrate efficacy of binimetinib and hydroxychloroquine in *KRAS*-driven lung carcinomas [79].

*ERBB2* (*HER2*) activating genetic events are characterized by significant diversity: they include exon 20 insertions and some rare mutations located within or outside the tyrosine kinase domain. They are detected in approximately 2−3% of LCs [5, 45, 80]. Small molecule inhibitors produced only limited benefit to patients with *ERBB2* (*HER2*)-mutated LC and, therefore, failed to receive FDA approval. In a study involving 90 previously treated patients with *ERBB2* (*HER2*) mutations, the RR for poziotinib was 28% and the median PFS approached 5.5 months [81]. Another poziotinib study included 80 treatment-naive LC patients and produced a somewhat higher RR (39%), but essentially the same median PFS (5.6 months) [82]. Importantly, different *ERBB2* (*HER2*) exon 20 insertion variants differed by their sensitivity to this compound [81, 82]. Similar to the situation with *EGFR* exon 20 insertions, an antibody-based approach turned out to be more efficient than specific targeting of mutated protein. Antibody-drug conjugate trastuzumab deruxtecan (T-DXd) was evaluated at two doses in previously treated subjects with *ERBB2* (*HER2*) mutations. It produced a 49% RR and a median PFS of 9.9 months at a tolerable dose of 5.4 mg/kg every 3 weeks, and, therefore, has received an approval for clinical use [17].

The mutational analysis of the listed above genes is less complicated than the evaluation of other LC-associated molecular targets (Table 3). Several allele-specific PCR kits are available for the detection of *BRAF* V600E and *KRAS* G12C mutations. *ERBB2* (*HER2*) exon 20 insertions are located within a relatively tiny genomic region and are also accessible to conventional DNA testing. Still, the use of NGS looks preferable, as it allows the detection of relatively rare targets, e.g., other than V600E drug-sensitizing substitutions in *BRAF* codon 600.

**Table 3:** Utility of different methods for detection of LC molecular targets

**Gene/Alterations**	**Variant-specific PCR**	**Sequencing of relevant DNA fragments**	**FISH**	**IHC**	**DNA-based NGS**	**RNA-based NGS**	**Other methods**
*EGFR* mutations in exons 18, 19 and 21	+	++	−	−	+++	+++	Capillary gel electrophoresis is reliable for detection of exon 19 deletions and insertions
*EGFR* exon 20 insertions	+	++	−	−	+++	+++	Capillary gel electrophoresis is reliable for detection of exon 20 insertions
*BRAF* mutations	+	++	−	−	+++	+++	
*KRAS* mutations	+	++	−	−	+++	+++	
*ERBB2* (*HER2*) exon 20 insertions	+	++	−	−	+++	+++	Capillary gel electrophoresis is reliable for detection of exon 20 insertions
*MET* exon 14 skipping mutations	+	−	−	−	+	+++	Comparison of expression of mutated vs. wild-type allele
*ALK* fusions	+	−	++	+	+	+++	PCR test for unbalanced 5’/3’-end expression
*ROS1* fusions	+	−	++	−/+	+	+++	PCR test for unbalanced 5’/3’-end expression (?)
*RET* fusions	+	−	−	−	+	+++	PCR test for unbalanced 5’/3’-end expression
*NTRK1−3* fusions	+	−	++	+	+	+++	PCR test for unbalanced 5’/3’-end expression
TMB	−	−	−	−	+++	+ (?)	
PD-L1 expression	−	−	−	+	−	−	

+++: highly reliable method; ++: method with some limitations; +: method with significant limitations; −: inappropriate method; (?): not enough evidence. The blank cells indicate that no methods other than the ones marked by “+” symbols have been sufficiently validated for the testing of this molecular marker. FISH: fluorescence in situ hybridization; IHC: immunohistochemistry; NGS: next-generation sequencing; TMB: tumor mutation burden

## Gene fusions resulting in the activation of RTKs

Although druggable gene fusions in LC had been discovered more than 15 years ago, their detection remains the most error-prone component of LC molecular diagnosis. Proper clinical management of RTK-rearranged LCs is associated with indeed dramatic improvement of patient outcomes (Table 1), with the median overall survival estimates usually exceeding 5-years threshold. RTK fusions are strongly associated with younger patients’ age, non-smoking history and female gender (Table 2). The cumulative frequency of RTK translocations varies between studies, as it depends on the age distribution within analyzed patient groups and the methodology of genetic analysis. It appears that approximately 1 out of 10 patients with LC carry actionable rearrangements, and this estimate is several times higher in LC non-smokers aged below 50 years.

*ALK* translocations, discovered in the year 2007, account approximately for 5% of lung carcinomas. The availability of an ALK inhibitor, crizotinib, facilitated immediate clinical studies on *ALK*-rearranged LCs, which were highly successful and quickly led to the approval of this drug [83]. There are several more modern ALK inhibitors, which are characterized by increased potency and good penetration into the brain. Alectinib and lorlatinib produced particularly impressive PFS estimates while given as first-line medication, with median OS not reached in both of these studies [24, 25, 84]. The prolonged survival of patients with *ALK*-rearranged LC signifies the emphasis on the quality of life of the treated patients, as many of them continue to work and manage regular social activities [12]. *ALK* rearrangements are particularly common in LC female patients aged below 40 years, and there are several reports describing childbirth during the treatment of *ALK*-driven LC [85].

Significant efforts have been invested in the development of methods for *ALK* translocation detection. Fluorescence in situ hybridization (FISH) was initially considered a “gold standard”, however, its utilization is complicated due to relatively high costs. Several antibodies for immunohistochemical (IHC) ALK detection produce acceptable balance between sensitivity and specificity, so they are permitted as a stand-alone test for ALK diagnostics [69, 86]. Still, there is a critical mass of evidence suggesting that “morphological” methods of ALK analysis are not sufficiently reliable and need to be replaced by the direct detection of translocations. Clinical studies demonstrate better outcomes in patients with *ALK* translocations confirmed by two independent methods as compared to subjects whose tumor was analyzed by a single IHC or FISH test [87, 88]. Furthermore, FISH and IHC are unable to detect translocation variants; this is a serious disadvantage at least for clinical studies, given that the identity of the translocation partner may influence outcomes of ALK-targeted therapy [89]. Indeed, some preclinical and clinical investigations revealed that so-called “short” ALK fusion variants (particularly, v.3 and v.5) are less sensitive to ALK inhibitors than the “long” fusion versions (v.1, v.2, etc.). In addition, *ALK* rearrangement variants may differ in their ability to acquire TKI-resistance via secondary mutation [90]. RNA sequencing is believed to be the best method for the analysis of actionable gene rearrangements, although it has not been subjected yet to rigorous multicenter validation studies [5, 86]. While discussing the advantages and disadvantages of PCR analysis, most relevant papers consider the so-called variant-specific PCR, i.e., the assays which are capable of detecting common translocations included in the corresponding kit or laboratory protocol [69, 86]. While conventional PCR procedure is unable to reveal yet unknown translocations, the test for unbalanced 5’/3’-end expression is potentially capable of detecting the entire spectrum of gene rearrangements. This assay relies on the fact that *ALK* rearrangement almost always results in the translocation of the kinase portion of the gene under control of a strong promoter belonging to the fusion partner. In fact, the same principle underlies the use of IHC for *ALK* testing, as the background expression of ALK in the lung tissue is low, and *ALK*-rearranged tumors are characterized by the elevated production of ALK kinase. In contrast to IHC, the 5’/3’-end expression PCR test utilizes the internal control, as it compares the amount of the kinase-specific RNA fragment towards the upstream *ALK* sequences. This procedure usually requires significant in-house validation, however, it appears to provide reliable results for *ALK* detection [91].

Approximately 1−2% of LCs carry *ROS1* rearrangements [70]. The majority of approved ALK inhibitors (crizotinib, ceritinib, brigatinib, lorlatinib) demonstrated clinical activity towards *ROS1*-rearranged tumors. Still, only crizotinib gained formal registration for the use in LC patients harboring *ROS1* translocations. In addition, *ROS1*-rearranged LC can be managed by NTRK/ROS1 inhibitors, entrectinib and repotrectinib [26–28]. Comprehensive studies on *ROS1*-driven LCs are complicated by the rarity of these events. Insufficient standardization of *ROS1*-testing procedures is another obstacle. ROS1 IHC testing can be used only as a prescreening procedure, and positive results always require a confirmation by an additional method [69, 70]. Unlike for *ALK*, the use of the comparative quantitation of 5’- and 3’-end portions of *ROS1* transcripts is compromised by the high background *ROS1* expression in the normal lung [69]. However, the diversity of *ROS1* fusions in LC is less pronounced as compared to other rearrangements, therefore, multiplexed variant-specific PCR appears to be a reasonable compromise [91]. As for other tyrosine kinase gene rearrangements, RNA sequencing is believed to be a preferential method of detection of *ROS1* fusions [5]. Similar to *ALK*, there are studies suggesting that distinct variants of *ROS1* fusions render distinct sensitivity to therapeutic ROS1 inhibition [92].

The frequency of *RET* fusions appears to be intermediate between estimates obtained for *ROS1* and *ALK* [71]. Unlike *ALK* and *ROS1*, *RET* fusions cannot be reliably detected either by IHC or FISH, therefore, relevant clinical studies relied on direct methods of identification of *RET* rearrangements. A number of multikinase inhibitors exert activity towards RET, however, their clinical use has been discouraged after RET-selective drugs, selpercatinib and pralsetinib, produced significantly better outcomes in prospective clinical trials [29, 30, 93, 94].

*ALK*-, *ROS1*- and *RET*-rearranged tumors have decreased expression of thymidylate synthase. Interestingly, a recent study suggested that this association is observed mainly in female but not male patients [71]. Low expression of thymidylate synthase may explain an increased sensitivity of *ALK*-, *ROS1*- and *RET*-driven cancers to pemetrexed [12, 71, 95–97].

*NTRK1*, *NTRK2* and *NTRK3* fusions are usually mentioned as agnostic targets. *NTRK1* and *NTRK3* translocations have been repeatedly described in LC patients, however, their cumulative frequency in LC falls below 1:500. The clinical features of *NTRK*-driven LCs are believed to be similar to other RTK-rearranged LCs, although rarity of these translocations precludes their detailed characterization [72]. Entrectinib and larotrectinib have been approved for the treatment of *NTRK*-driven malignancies irrespective of their histological origin [98, 99]. Separate analyses for *NTRK*-rearranged LC patients have been recently published for both these drugs, and they produced highly encouraging results [31, 32].

Activating *CLIP::LTK* fusions are exceptionally rare. They are associated with LC sensitivity to lorlatinib [100].

## *MET* exon 14 skipping mutations

Although LC-specific *MET* alterations are classified as mutations, their diversity, significance and preferred methods of detection place them closer to RTK rearrangements than to hot-spot alterations affecting *EGFR*, *ERBB2* (*HER2*), *BRAF* or *KRAS* oncogenes. LC-inducing *MET* mutations lead to exon 14 skipping, which eventually results in the significant increase of the half-life of MET receptor. Despite the clear-cut effect of *MET* alterations on protein stability, IHC screening for exon 14 skipping mutations turned out to be unreliable [67]. DNA-based detection of *MET* alterations is problematic because the position of the mutation is highly variable and cannot be reliably covered by conventional DNA sequencing [101]. *MET* transcripts lacking exon 14 can be detected by allele-specific PCR targeted towards the exon 13−15 junction in the cDNA, however, this approach is error-prone because some carcinomas may demonstrate low-abundance *MET* exon 14 skipping message. Reliable detection of oncogenic *MET* mutations can be accomplished either by RNA sequencing or by quantitative comparison of the expression of mutated versus wild-type transcripts [101, 102].

The distribution of *MET* mutations is clearly distinct from other alterations detectable by RNA-based methodologies. While RTK rearrangements are associated with young patients’ age, almost all subjects with *MET* exon 14 skipping mutations are older than 70 years [67, 68, 102]. MET-specific inhibitors capmatinib and tepotinib have received FDA approval for the treatment of cancers harboring this category of *MET* alterations [22, 23]. Crizotinib, which is approved for the treatment of *ALK*- and *ROS1*-driven carcinomas, was historically developed as a MET inhibitor and, expectedly, demonstrated clinical activity towards *MET*-mutated carcinomas [103]. Its use may be considered in instances of compromised access to capmatinib or tepotinib.

## Amplification and overexpression of druggable kinase-encoding genes

LC oncogene amplifications are particularly well described for *MET* and *ERBB2* (*HER2*) oncogenes. Clinical research of druggable gene extra copies is complicated by the ambiguities in their detection [5]. FISH analysis is a “gold standard” method for identifying gene amplifications. However, it requires the use of an individual test for each given gene, so it cannot be applied to rare genetic events, especially if their clinical significance is not firmly established. Furthermore, while a strong relationship between *ERBB2* (*HER2*) amplification and overexpression is well known for breast cancer [104], these tests may not be interchangeable for other tyrosine kinases or other cancer types. Indeed, some gene extra copies are not accompanied by the increased production of corresponding transcripts, and overexpression of several tyrosine kinases may occur in the absence of detectable DNA alterations. DNA-based NGS assays often account for oncogene amplifications, although they remain insufficiently validated in this respect. *MET*-amplified LCs demonstrated some degree of sensitivity to MET inhibitors within clinical trials, however the predictive value of *MET* extra copies was lower as compared to *MET* exon 14 skipping mutations [22, 105, 106]. *MET* amplification is a common root of tumor escape from inhibition of other tyrosine kinases, which is particularly well exemplified for EGFR TKIs. Consequently, the ability of MET-specific drugs to combat the resistance to various targeted drugs is a subject of intensive investigations [107]. Approximately 1% of lung carcinomas demonstrate amplification and overexpression of *ERBB2* (*HER2*) oncogene. These LCs appear to be reasonably responsive to conventional and novel anti-HER2 drugs [80, 108, 109]. There are examples of response of ALK-overexpressing LCs to crizotinib despite the absence of *ALK* rearrangements [110]. Actionable activating events in the MAPK signaling pathway are almost always mutually exclusive. Perhaps, it is justified to consider tyrosine kinase overexpression mainly in the tumors lacking known LC-associated oncogenic mutations. Furthermore, the profound genetic and expression analysis is particularly justified in tumors with acquired resistance to targeted treatment, given that escape from targeted therapy is very often attributed to the activation of collateral signaling cascades [111].

## Tumor mutation burden

Tumor mutation burden (TMB) is an integral characteristic of tumor genome accounting for the overall number of mutations. TMB was initially evaluated by whole exome sequencing, and only non-synonymous mutations were taken into consideration. High number of coding mutations is associated with increased tumor immunogenicity, therefore the existence of correlations between high TMB and increased responsiveness to ICIs fits to the current knowledge [112, 113]. Some important nuances may compromise the use of TMB in the clinical routine. First of all, TMB strongly correlates with smoking history in LC patients, so the self-reported data on cigarette use may serve as an easily accessible and reasonably reliable surrogate of TMB [114]. TMB by definition requires the use of NGS, which is often time-consuming. Many multigene sequencing services offer evaluation of TMB based on the analysis of several hundred genes; in contrast to whole exome sequencing, these services often consider both non-synonymous and synonymous mutations [112]. As for many continuous variables, the choice of the most clinically relevant TMB threshold presents a challenge. Different studies applied different cut-offs to discriminate between tumors with high and low TMB [112, 113]. It is necessary to keep in mind that some NGS tests are limited by LC-specific actionable genes [6], and these mini-panels, being non-expensive and reliable for the analysis of main druggable mutations, are not representative for TMB calculation. The established methodologies of TMB analysis involve DNA-based NGS. However, RNA-based NGS has an obvious advantage for LC testing, as it is capable of identifying both oncogene-activating mutations and kinase gene rearrangements. There are studies demonstrating that TMB can be reliably calculated using RNA sequencing data [115].

## PD-L1

The analysis of PD-L1 status has been incorporated into the decision-making process for administration of several ICIs. In general, lung adenocarcinomas, which lack activating mutations in kinase genes, demonstrated a consistent association between the level of PD-L1 expression and the probability of response to ICI therapy [116]. Still, the reliance on PD-L1 testing is compromised by several factors. Some ICI studies considered the percentage of IHC-stained tumor cells, while other clinical trials analyzed the combined PD-L1 expression status of both tumor and immune cells. There is no biological rationale justifying these differences as well significant interstudy variations in the ranking of thresholds. It is firmly established that LCs with high PD-L1 expression (greater than or equal to 50% tumor cells) have a significantly increased probability of deriving benefit from ICI therapy. However, the value of intermediate PD-L1 scores (1−49%) is less reproducible, and even PD-L1 negative tumors have some probability of responding to ICI. PD-L1 status is mainly relevant to the choice of first-line therapy, as it allows for selection of patients who may be treated by ICIs without cytotoxic drugs. Multiple clinical trials demonstrated that the administration of ICI in combination with chemotherapy or after tumor progression on chemotherapy does not require determination of PD-L1 status [116–119]. Interestingly, cytotoxic drugs are able to induce PD-L1 expression *in vivo*, as well as utilize other roots of making tumors susceptible to ICIs [120, 121].

PD-L1 analysis is a mandatory part of LC management. While all other molecular targets can be determined by genetic testing, particularly by NGS, PD-L1 expression is currently evaluated by IHC and, therefore, requires an additional processing of tumor samples. RNA-based analysis of *PD-L1* expression level demonstrates satisfactory results and, hence, may deserve consideration as a promising predictive marker [122, 123]. Furthermore, a history of heavy smoking or never-smoking is highly predictive for response versus non-response to ICI, so this information can be used in the decision-making process for patients with unknown PD-L1 staining score [114]. Importantly, the majority of never-smokers have actionable mutations in the kinase genes [124], so their PD-L1 status may be considered only in combination with comprehensive genetic testing.

## Choice of adjuvant therapy based on mutation profiling

Historically, trials on adjuvant cancer therapy dealt with significant levels of uncertainty. Indeed, most therapeutic schemes are associated with response rates around or below 50%, and the individual probability of benefit for a given patient with invisible cancer cannot be reliably assessed. Conventional adjuvant treatments involve either a short-term administration of chemotherapy, or a prolonged use of perfectly tolerable drugs (e.g., endocrine or HER2-directed therapy for breast cancer). In contrast to the above examples, some druggable oncogene mutations are associated with a nearly 100% probability of tumor response. Not surprisingly, all properly designed adjuvant trials involving these highly efficient drugs demonstrated evident prolongation of the disease-free interval with a minimal number of relapses occurring during this treatment. However, their influence on the cure rates is less obvious, because in a subset of patients, this therapy only delayed but did not prevent disease recurrence [125]. There are several issues which are extremely difficult to address. The optimal duration of adjuvant therapy is unknown. On one hand, several clinical trials demonstrated an increased rate of relapses occurring soon after the completion of adjuvant therapy [125]. These data indicate that mutation-tailored drugs do not always kill all residual cancer cells, but rather prevent their expansion. Consequently, some subjects may require life-long exposure to a targeted drug. On the other hand, some clinical studies revealed comparable outcomes for short-term versus long-term targeted therapy [126]. Indeed, if a particular drug is capable of eliminating all transformed cells carrying the target, there is no need for a prolonged use of this compound, especially given that the total tumor burden is low after surgical removal of visible cancer lumps. There are no reliable animal models which allow the resolution of the above controversy. For the time being, the duration of adjuvant targeted therapy is influenced mainly by consideration of its adverse effects and costs. The analysis of circulating tumor DNA (ctDNA) may, in theory, assist the personalization of adjuvant administration of mutation-tailored drugs [127–129]. However, ctDNA is not sufficiently informative in patients with low overall tumor burden and in subjects whose tumors respond to the treatment at the time of blood-take [127, 130, 131].

The rarity of druggable genetic alterations and the continuous emergence of new targeted drugs also present a significant obstacle for relevant adjuvant trials. *EGFR* mutations are significantly more common than other molecular targets, however, even for them the progress towards adjuvant therapy approval has taken more than a decade [132]. Relevant investigations on other LC-associated molecular events are more problematic. For the time being, the results of adjuvant alectinib study in *ALK*-rearranged patients have been released, and they are generally similar to respective *EGFR* trials [133]. Comprehensive analysis of ROS1-targeted drugs is complicated even for metastatic patients, and the same is true for several other rare target-drug matches. Perhaps, some extrapolation of the results obtained upon adjuvant trials on EGFR and ALK inhibitors is feasible, especially given that at least short-term postsurgical administration of TKI is not associated with significant adverse events.

## Other potentially relevant somatic mutations and expression markers

*MAP2K1* (*MEK1*) activating mutations are detected in less than 1% of LCs. They are characterized by several hot-spots and, therefore, can be examined both by conventional techniques and by NGS. A few instances of responses of LC and non-LC *MAP2K1*-mutated tumors to therapeutic MEK inhibitors have been described in literature [134–136].

*TP53* mutations occur in about a half of LCs. Their presence is consistently associated with poorer response to TKI treatment [89, 137, 138]. However, there is no modification of the treatment allowing to customize drug choice to wild-type versus mutated *TP53*, therefore, the knowledge on *TP53* mutation status is currently not actionable. It is necessary to keep in mind that the detection of *TP53* mutation in the tumor tissue does not necessarily indicate the somatic origin of this variant. Indeed, non-smoking young-onset patients with *TP53* mutations are strongly advised to undergo testing for Li-Fraumeni hereditary cancer syndrome [139].

*KEAP1* and *LKB1* (*STK11)* mutations occur in approximately 20% of lung carcinomas. They are associated with poor prognosis and low tumor responsiveness to chemotherapy and immune therapy. This trend has also been observed in some studies on inhibitors of signaling cascades. For example, a clinical trial on sotorasib demonstrated a numerically lower response rate for *KEAP1* mutated versus wild-type LC [140]. However, the presence of *KEAP1* or *STK11* mutations does not influence treatment choice, therefore, these genes are not included in the standard diagnostic panels [141, 142].

*PIK3CA* somatic mutations activate catalytic subunit of the phosphoinositide 3-kinase (PI3K). They play an oncogenic role by increasing cell survival and modulating other cancer-related pathways. While PIK3CA inhibition is utilized in breast cancer treatment, this therapeutic option failed to demonstrate efficacy in a LC clinical trial [143].

Although the inhibitors of signaling cascades significantly prolong survival of cancer patients, none of them are truly curative because they are unable of achieving a complete elimination of malignant cells. The concept of persistence of residual cancer clones often refers to so called cancer stem cells (CSCs), which resist conventional treatment and secure tumor repopulation. These CSCs express unique markers, which can be used for their identification and targeting. A number of experimental drugs aimed at specific suppression of CSCs are currently at various stages of development [144].

## Germline mutations

LC is rarely associated with common hereditary cancer syndromes, so germline DNA testing is not a part of routine LC management [145]. Most LCs have an obvious cause, i.e., smoking, and familial clustering of this disease is rare even in young-onset cases. Still, there are notable examples of LC attributed to inheritance of pathogenic variants.

Li-Fraumeni syndrome is the most established example of genetic predisposition to LC. Li-Fraumeni syndrome is caused by germ-line mutations affecting *TP53* gene. It usually manifests by childhood tumors, brain malignancies, sarcomas, breast cancers and carcinomas of the lung [145, 146]. Almost all tumors obtained from Li-Fraumeni syndrome-related LC patients contain *EGFR* mutations [147].

*EGFR* T790M mutations have been discovered via the analysis of LCs with acquired resistance to gefitinib and erlotinib. Subsequent studies revealed that some patients contain this variant in a germline DNA, and it causes clustering of LC cases within pedigrees. Oxnard et al. [148] demonstrated that the majority of *EGFR* T790M-related familial LCs are attributed to a relatively recent North American founder mutation, while the worldwide contribution of this allele in LC incidence is very low [148–150]. Several case reports described other LC-predisposing germline pathogenic variants in the *EGFR* gene [151, 152].

*BRCA1* and *BRCA2* genes are recognized for their role in predisposing to breast, ovarian, pancreatic and prostate cancer. Recent studies suggest a significant level of promiscuity for these and some other tumor-associated genes, i.e., their moderate association with a wide variety of cancer types [145]. Elevated frequencies of *BRCA1/2* pathogenic variants in LC patients have been described in several studies [153, 154]. In contrast to breast and ovarian cancers, *BRCA1/2* germ-line alterations, although being presumably a cause of LC disease in mutation carriers, are not always accompanied by the loss of the remaining *BRCA1/2* allele, so they are not necessarily associated with tumor sensitivity to PARP inhibitors [155].

## LC diagnostic pipeline

**Figure 1:**
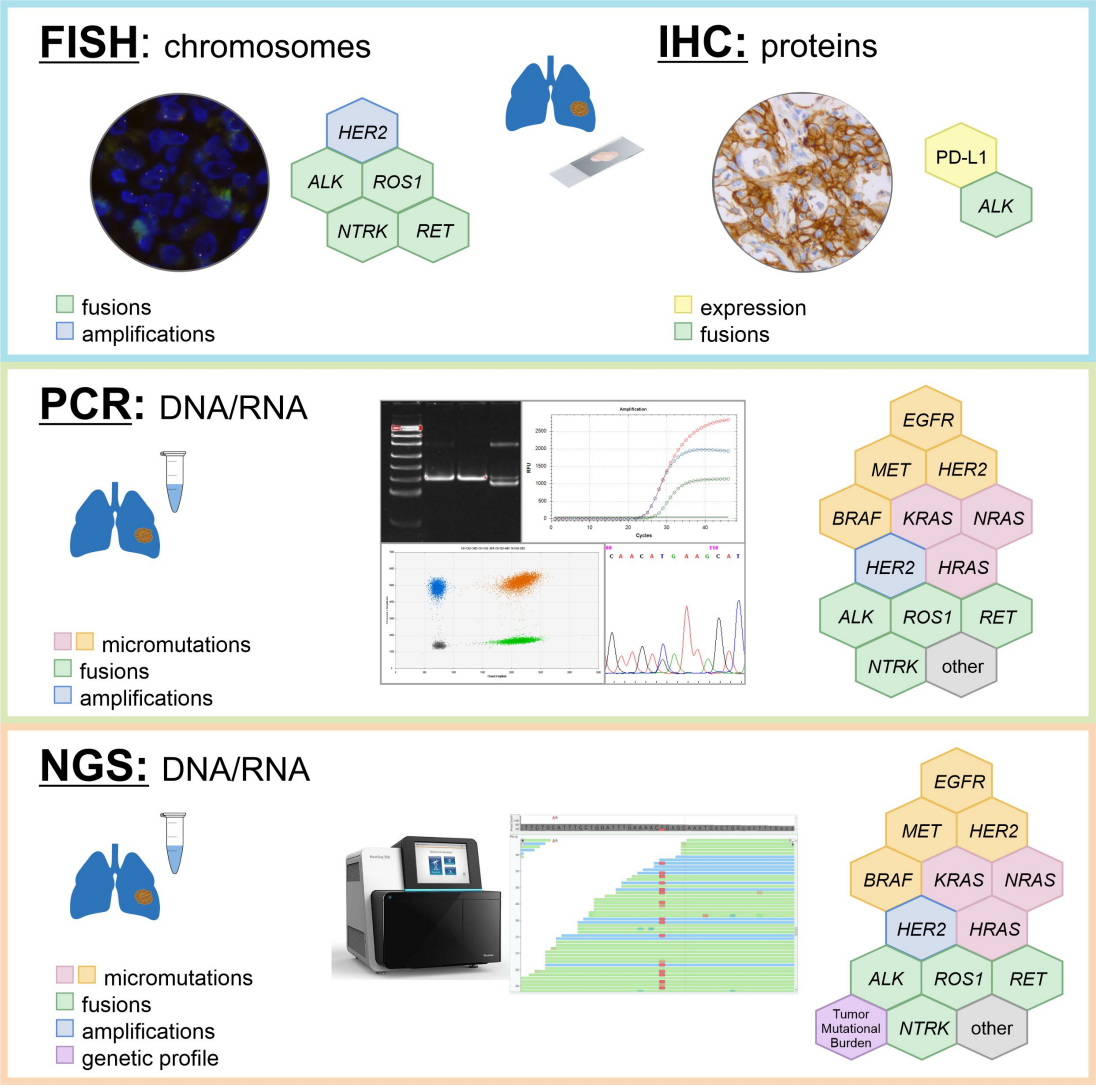
Detection of LC molecular targets by different methods

In an ideal situation, all patients with non-small cell LC have to be subjected to a comprehensive analysis of all druggable genes, evaluation of TMB and examination of PD-L1 status immediately after morphological analysis (Table 3, Figure 1). In theory, this can be achieved by combination of RNA sequencing and PD-L1 IHC testing, however, even advanced cancer centers currently prefer the sequential use of DNA-based and RNA-based NGS [5]. Turn-around-time is critical for proper LC management [3]. Clinical studies indicate that superior results can be achieved only in patients who received their targeted therapy in the first-line setting, which is compatible with the testing procedures taking no more than 10 working days [156]. Although NGS technologies are increasingly positioned as the “one-for-all” diagnostic approach, their time requirements in a real clinical world have not been adequately assessed yet. In addition, local availability of targeted drugs, costs of LC mutation analysis and convenience significantly influence the choice of preferred diagnostic attitudes [5, 59, 157].

*EGFR* mutations are particularly frequent in LC patients of Asian ancestry, therefore, rapid single-gene *EGFR* testing may be a feasible initial approach to this category of subjects [158]. Furthermore, EGFR inhibitors have already become available as generic compounds, therefore, they are better available than other drugs in countries with limited resources [159]; consequently, NGS is unlikely to fully replace conventional LC molecular testing in the next few years. Many hospitals continue to utilize IHC and/or FISH for the analysis of *ALK*, *ROS1* and *NTRK1-3* gene rearrangements for the sake of convenience [6]. Despite the fact that these tests can be performed in local laboratories, they have imperfect reliability and may compromise timing or tissue availability for comprehensive LC profiling [3, 4]. While PCR analysis of hot-spot mutations in actionable genes seems to be reasonably justified at least in some circumstances, the use of surrogate methods, like IHC or FISH, appears to be indeed disadvantageous and may need to be discouraged in the future. Some studies have reported the use of laboratory-developed protocols for the detection of druggable gene translocations, which combine multiplexed variant-specific PCR with the test for 5’/3’-end unbalanced expression. This approach may be viable, because it is characterized by short turn-around-time, cost-efficiency and minimal tissue requirements [71, 73, 91].

The discussion on advantages and disadvantages of RNA NGS-based comprehensive molecular profiling versus local single-gene testing requires thoughtful and delicate adjustment to local circumstances, particularly speed and pricing of the available services, actual accessibility of targeted drugs, human resources, hospital and laboratory infrastructure, preferences of doctors and patients, regulatory issues, etc. The performance of diagnostic services has to be controlled against the “gold standard” facilities: for example, pathological units may demonstrate significant interlaboratory variations with regard to their reliability for detecting gene fusions [69, 70, 86]. Cost considerations are essential, given that the price for PCR in some countries may be as low as a few EURO or USD per reaction, while IHC, FISH and NGS generally require 1−2 orders of magnitude higher expenses [73]. On the long-term run NGS will certainly replace other techniques of LC molecular analysis, however, for the time being, many cancer hospitals are looking for a balance between high-throughput and conventional methods of genetic testing [3–6].

## Conclusions

Targeted drugs render remarkable survival benefit to LC patients with druggable genetic alterations. This advantage is generally more pronounced for gene fusions than for mutation-tailored therapies. The majority of targeted compounds, for example, EGFR, BRAF and MET inhibitors, as well as molecules directed towards rearranged kinases, are particularly effective when administered in the very beginning of the treatment of metastatic disease. Proper LC diagnosis requires comprehensive examination of approximately a dozen genes within two-week interval, therefore, simultaneous molecular testing is strongly preferred over the sequential approach. “Morphological” methods of gene analysis, e.g., IHC or FISH, are insufficiently sensitive and specific when compared to DNA/RNA-based detection of genetic alterations. Mutation- and variant-specific PCR kits are reliable only for “positive findings”, while the “negative results” may be attributed to their intrinsic inability of detecting uncommon molecular targets. RNA-based NGS is gradually becoming a dominating technology in comprehensive LC testing. The concept of the adjuvant use of targeted drugs needs to be thoughtfully discussed, because conventional adjuvant clinical trials cannot efficiently account for rare genetic alterations and continuous increase of the spectrum of novel inhibitors of signaling cascades.

## Abbreviations

CSCs: cancer stem cells
ex19del: exon 19 deletion
FISH: fluorescence in situ hybridization
ICIs: immune checkpoint inhibitors
IHC: immunohistochemical or immunohistochemistry
LC: lung cancer
NGS: next-generation sequencing
OS: overall survival
PFS: progression-free survival
RR: response rate
RTK: receptor tyrosine kinase
TKI: tyrosine kinase inhibitor
TMB: tumor mutation burden

## Declarations

### Copyright

© The Author(s) 2024.
